# Risk Factors and Prediction Models for Early and Long-Term Mortality Following Left Ventricular Assist Device Implantation

**DOI:** 10.3390/jcm15187168

**Published:** 2026-09-15

**Authors:** Balakrishnan Mahesh, Dongping Du, Kenny Nguyen, Corrine Corrina Hartford, Swarna Mahesh, Nandini Nair

**Affiliations:** 1 Heart and Vascular Institute, Penn State Milton S. Hershey Medical Center, Hershey, PA 17033, USA; bmahesh@pennstatehealth.psu.edu; 2 Department of Industrial, Manufacturing and Systems Engineering, Texas Tech University, Lubbock, TX 79409, USA; dongping.du@ttu.edu; 3 Department of Surgery, PSU College of Medicine, Penn State Milton S. Hershey Medical Center, Hershey, PA 17033, USA; kennyknguyen5@gmail.com (K.N.); chartford@pennstatehealth.psu.edu (C.C.H.); 4 Department of Medicine, Division of Cardiology, Penn State Milton S. Hershey Medical Center, Hershey, PA 17033, USA; smahesh555000@gmail.com

**Keywords:** risk factors, machine learning, risk assessment, ventricular assist device, extracorporeal membrane oxygenation

## Abstract

**Introduction:** Changes in the United Network for Organ Sharing (UNOS) allocation system, coupled with declining donor availability, have contributed to a rising proportion of durable left ventricular assist device (LVAD) implantations, particularly among patients with prior cardiac surgeries and those requiring multiple concomitant intracardiac procedures. As the complexity of LVAD candidates increases, understanding peri/postoperative determinants of survival becomes critical. We sought to identify risk factors associated with short- and long-term mortality following LVAD implantation. **Methods:** We analyzed 22,467 adults (18–80 years) who underwent LVAD implantation between January 2013 and June 2023 using data from the INTERMACS registry. A FasterRisk-derived risk score was developed for 90-day mortality prediction, and a Cox proportional hazards model was constructed to evaluate long-term post-implant survival. **Results:** Predictors of 90-day mortality included advanced age, preoperative extracorporeal membrane oxygenation (ECMO), LVAD exchange, prior cardiac surgery, and concomitant surgery. A 90-day mortality score incorporating pre-implant ECMO, prior cardiac surgery, concomitant surgery, and age demonstrated c-statistic 0.69 in training and 0.67 in testing. The Cox regression model identified advanced age, preoperative ECMO, previous cardiac surgery, planned concomitant RVAD, concomitant surgery, IABP, CVA and postoperative dialysis as significant predictors of long-term mortality (c-index 0.72 training; 0.70 testing). **Conclusions:** A cross-validated machine learning-based risk model for 90-day mortality following LVAD implantation and a Cox regression model for long-term survival may support improved risk stratification and perioperative management in LVAD patients.

## 1. Introduction

In 2018, the United Network for Organ Sharing (UNOS) revised the heart allocation system, replacing the 2006 policy with more objective, hemodynamic-based status criteria [1,2,3,4,5,6]. As candidates with advanced comorbidities increase and donor availability declines, the use of durable left ventricular assist devices (LVADs) for bridge-to-transplant and destination therapy has grown, with survival now approaching that of transplantation [2,3,4]. Earlier LVAD implantation may reduce hospitalizations and limit progression of end-organ dysfunction. Despite advances in contemporary devices, early postoperative mortality particularly within 90 days remains significant. This study evaluated how previous and concomitant cardiac surgeries influence short- and long-term outcomes after LVAD implantation, identified additional independent predictors of postoperative risk, and assessed the performance of two prognostic approaches, namely the FasterRisk machine learning model for 90-day mortality and a Cox regression model for long-term survival.

## 2. Materials and Methods

Between January 2013 and June 2023, 22,467 LVAD implants in adults aged 18–80 were analyzed using INTERMACS data processed in SPSS 29.0 and Python version 3.11 [6]. Continuous variables were compared using *t* tests or Mann–Whitney U tests, and categorical variables were assessed with chi-square tests, with significant predictors (*p* < 0.1) entered into multivariable logistic regression and incorporated into the FasterRisk model to generate a cumulative early mortality risk score [6]. Five-fold cross-validation identified the optimal number of predictors for the early mortality score with highest c statistic and least model complexity, which was then evaluated using ROC analysis, calibration, and risk stratification. Long-term survival was assessed with Kaplan–Meier with log-rank testing including multivariable Cox regression proportional hazards model (confidence interval of 95%). Statistical significance was defined as *p* < 0.05. The INTERMACS database is a public database with no patient identifiers; hence it is exempt from IRB. There was no direct patient recruitment or participation in this study and hence no consents were needed. This study is a retrospective de-identified data review with no identifiers of any sort. The INTERMACS data was obtained by the Data Use Agreement for INTERMACS at Penn State College of Medicine/Department of Surgery and was IRB exempt as it is a decoded database with no patient identifiers.

### 2.1. Study Cohort Derivation

**Figure 1:**
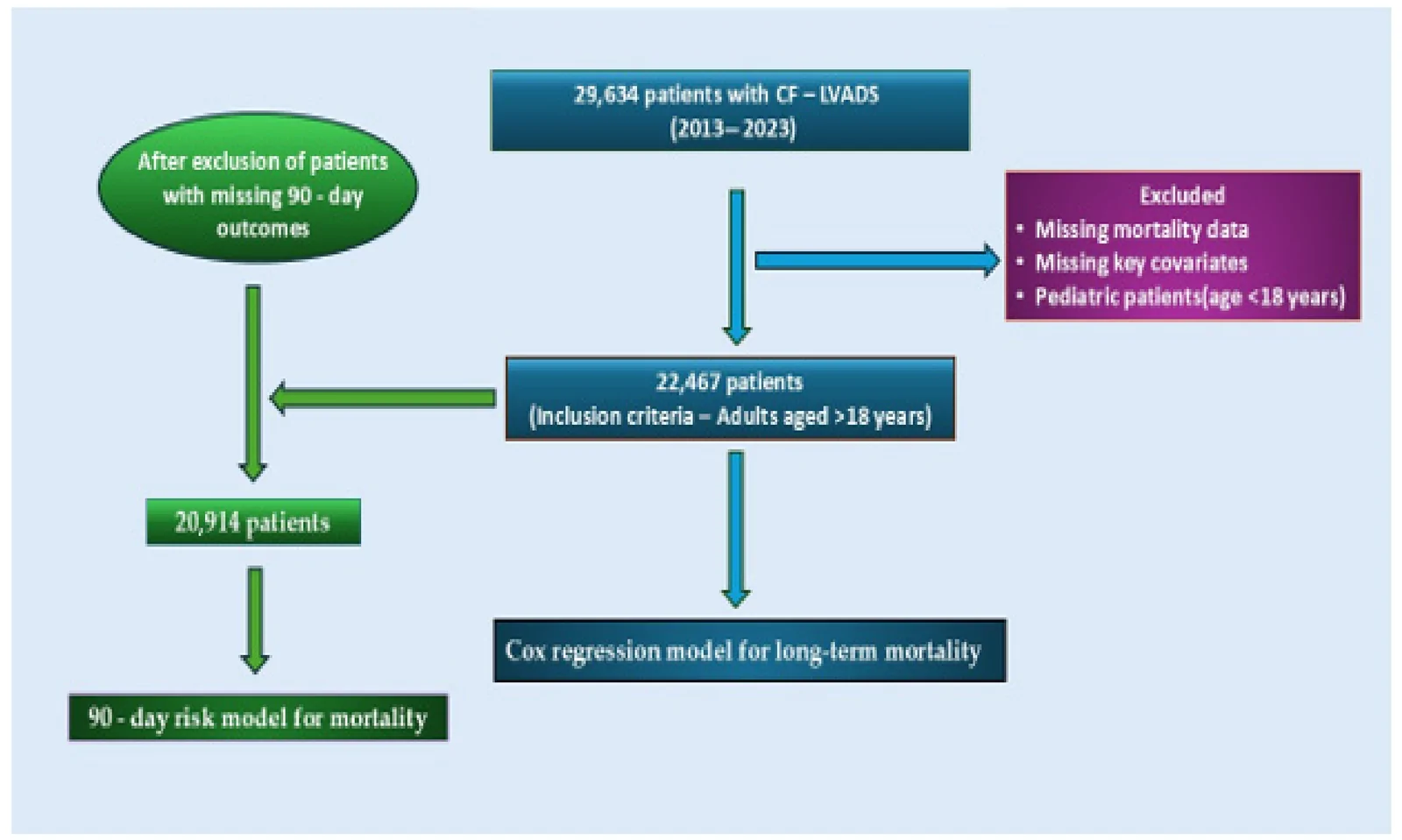
Flow Chart for study cohort derivation denotes the derivation of the cohorts used for 90-day risk model for short-term mortality and the Cox regression model for long-term mortality.

This study uses the INTERMACS database; hence, it is a retrospective analysis. This is not a prospective enrollment study. Figure 1 shows a flow diagram describing the study cohorts. The study cohort included all adults in the INTERMACS registry who underwent durable LVAD implantation between 2013 and 2023 (*n* = 29,634). After excluding patients younger than 18 years and those with missing mortality data or essential covariates, 22,467 patients remained for derivation of the Cox regression model for long-term mortality. From this group, patients lacking 90-day outcome data were further excluded, yielding a final cohort of 20,914 patients for development of the 90-day mortality risk model. The index event was the first recorded primary durable LVAD implantation. The primary outcome was 90-day all-cause mortality as the primary endpoint and long-term mortality as the secondary endpoint.

### 2.2. Development of Risk Prediction Model Using FasterRisk for 90-Day Mortality After LVAD Implantation

The cohort included 20,914 LVAD recipients, with 2221 deaths and 18,693 survivors by 90 days. Patients who died were older (60 ± 12 vs. 56 ± 13 years). Variables associated with 90-day mortality on univariate analysis (*p* < 0.10) were advanced to multivariable modeling, and those retaining *p* < 0.10 were considered for the risk score based on odds ratios and contribution to discrimination. The dataset was split into 80% training and 20% testing subsets, and all analyses were performed in Python using scikit-learn and FasterRisk. Survival analysis and LASSO-penalized Cox regression were done using the lifelines (version 0.30.3) and scikit-survival (version 0.28.0) packages. The 90-day risk score, univariate analysis, and multivariable analysis were performed using the FasterRisk (version 0.1.10), statsmodels (version 0.14.6), scikit-learn (version 1.9.0) packages.

### 2.3. Evaluation of Model Performance for 90-Day Mortality

Calibration was evaluated using curves comparing mean predicted and observed 90-day mortality across quantile bins, where closer alignment to the diagonal reflects better calibration. The bar plots show predicted versus observed mortality across five risk categories (very low to very high) to assess calibration consistency and stability across strata.

### 2.4. Risk Stratification for Long-Term Survival

LASSO-penalized Cox regression was applied within a cross-validation framework to select features, using the 1-standard-error rule to favor a more parsimonious model. Variables with non-zero coefficients at the chosen penalty level were then entered into a multivariable Cox proportional hazards model for final estimation. Individual risk scores were generated from the refitted Cox model and used to stratify patients into quantile-based risk groups, with observed survival estimated by Kaplan–Meier curves and model-based survival derived from predicted functions. Discrimination was evaluated through separation of Kaplan–Meier curves, log-rank testing, and comparison of median survival times with confidence intervals. This two-stage process produced a sparse, interpretable, and clinically meaningful risk stratification model.

### 2.5. Construction of Decision Analysis Curves

Decision curve analysis (DCA) was performed to assess the clinical utility of the 90-day risk model and the long-term risk model. Net benefit across clinically relevant threshold probabilities was calculated using the Python d curves package. A decision curve was constructed to compare the net benefit of the 90-day risk score with treat-all and treat-none strategies. DCA was conducted at 1, 3, and 5 years after implantation using predicted mortality derived from the long-term Cox risk model. The model was compared with the default strategies of treating all patients versus treating no patients.

## 3. Results

### 3.1. Baseline Characteristics of the Patients in the Study Cohort

As this was a retrospective study using a publicly available database, individual patient-level indications for LVAD implantation were not available. According to INTERMACS reports, approximately 35% to 45% of LVAD recipients have ischemic cardiomyopathy and 55% to 65% have non-ischemic cardiomyopathy. Commonly reported comorbidities in the INTERMACS population include diabetes mellitus, hypertension, atrial fibrillation, and chronic kidney disease; however, the prevalence of these conditions in our specific cohort could not be determined. Additionally, the standardized INTERMACS dataset does not capture heart failure treatment duration before LVAD implantation or details of concomitant medical therapy. In clinical practice, durable LVAD implantation is generally indicated for patients with advanced heart failure refractory to guideline-directed medical therapy to improve functional status, quality of life, and survival. To provide further clinical context, available baseline patient characteristics of our cohort are shown in Table 1.

Table 2 shows the distribution of the various concomitant cardiac surgeries performed during the period of investigation in the study cohort. Surgery using minimally invasive techniques was performed in 852 patients [3.8%]. During surgery, 1009 patients (4.5%) were transitioned from preoperative ECMO to durable LVAD support. Devices implanted included durable LVADs in 20,636 patients (91.8%), durable LVAD plus temporary RVAD in 1335 (5.9%)**,** and total artificial hearts in 466 (2.1%). Postoperatively, 785 patients (3.5%) required ECMO, and 4868 (21.7%) underwent concomitant cardiac surgery.

### 3.2. Postoperative Complications

In the postoperative period, 1730 patients (7.7%) required re-exploration for bleeding, and subsequently 249 patients (1.1%) developed a delayed pericardial effusion requiring pericardiocentesis. ECMO support was needed in 785 patients (3.5%). Other major complications included gastrointestinal bleeding in 4855 patients (21.6%), stroke in 3450 patients (15.4%), and dialysis in 4189 patients (18.6%). By 90 days, 2221 patients (9.9%) had died.

### 3.3. Predictors of 90-Day Mortality

#### 3.3.1. Univariate Analyses

A total of 16 univariate preoperative, intraoperative, and postoperative variables were associated with 90-day mortality. Preoperative predictors included increasing age, LVAD exchange, preoperative ECMO support, elevated right atrial and wedge pressures. Significant intraoperative factors were device type (temporary LVAD, RVAD) concomitant cardiac surgery, and LVAD explantation. Postoperative predictors included RVAD, IABP, or ECMO support, reoperation for bleeding within and after 48 h, prolonged ventilation, GI bleeding, stroke, and long-term hemodialysis (Table 3A).

#### 3.3.2. Multivariate Analyses

Of the 16 univariate variables tested, 11 variables in multivariable analysis remained significant predictors of 90-day mortality (*p*-value cutoff = 0.1). The preoperative variables that remained significant were age, LVAD exchange, and preoperative ECMO support. LVAD exchange had the maximum impact with an odds ratio of 3.13 (*p* = 0.008). The intraoperative variable that had maximum significance in multivariable analyses was temporary RVAD with a significant odds ratio of 5.07 (*p* < 0.001). Postoperative variables that were significant were ECMO support, prolonged ventilation, stroke, return to operating room for bleeding, and long-term hemodialysis (Table 3B).

#### 3.3.3. Risk Score for Predicting Early Mortality

Seven preoperative variables with assigned point values (Table 4A) were used to generate a total risk score ranging from zero to 12. Younger age and LVAD implantation were associated with lower scores, reflecting their protective effect. Conversely, higher scores corresponded to increased mortality risk (Table 4B). The percentage mortality shown in Table 4B was derived using the logistic function P(mortality) = 1/(1 + exp(−(score-9)/2.734).

**Figure 2:**
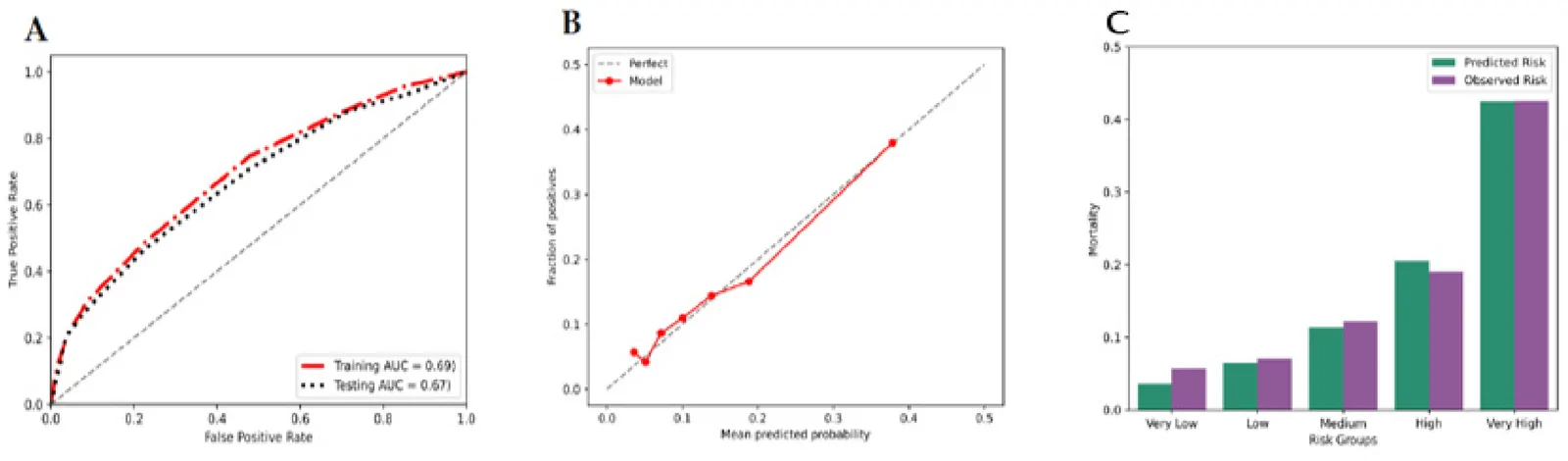
Performance of the 90-day risk score. Performance and calibration of the 90-day mortality risk prediction model. (**A**) Receiver operating characteristic (ROC) curves demonstrating the discriminative performance of the 90-day mortality risk score in the training and test cohorts. The red line depicts training ROC, and the black dotted line shows the testing data. The gray dashed line shows the reference points where AUC = 0.5. The model achieved an area under the curve (AUC) of 0.69 in the training set and 0.67 in the test set. The diagonal dashed line represents no-discrimination of performance. (**B**) Calibration plot for the test cohort comparing predicted and observed 90-day mortality probabilities. The dashed gray line indicates perfect calibration, while the red line represents model performance across risk strata. (**C**) Comparison of predicted and observed mortality rates across risk categories (very low, low, medium, high, and very high) in the test cohort. Predicted and observed mortality rates demonstrated good agreement across risk groups, supporting the model’s calibration and clinical utility for risk stratification. Abbreviation: AUC, area under the receiver operating characteristic curve; ROC, receiver operating characteristic.

Prior cardiac surgery, concomitant RV support, pre ECMO and concomitant surgery were significant. The c-statistic was 0.69 in the training cohort and 0.67 in the testing cohort (Figure 2A). The calibration curve and risk stratification plots are represented in Figure 2B and Figure 2C, respectively, which show good performance of the model. The risk stratification begins with preoperative variables that provide an indication of the preoperative mortality risk percentage. By incorporating readily available preoperative variables, this scoring system provides a practical tool for estimating mortality risk and supporting clinical decision-making, patient counseling, and perioperative planning.

### 3.4. Predictors of Long-Term Survival

**Figure 3:**
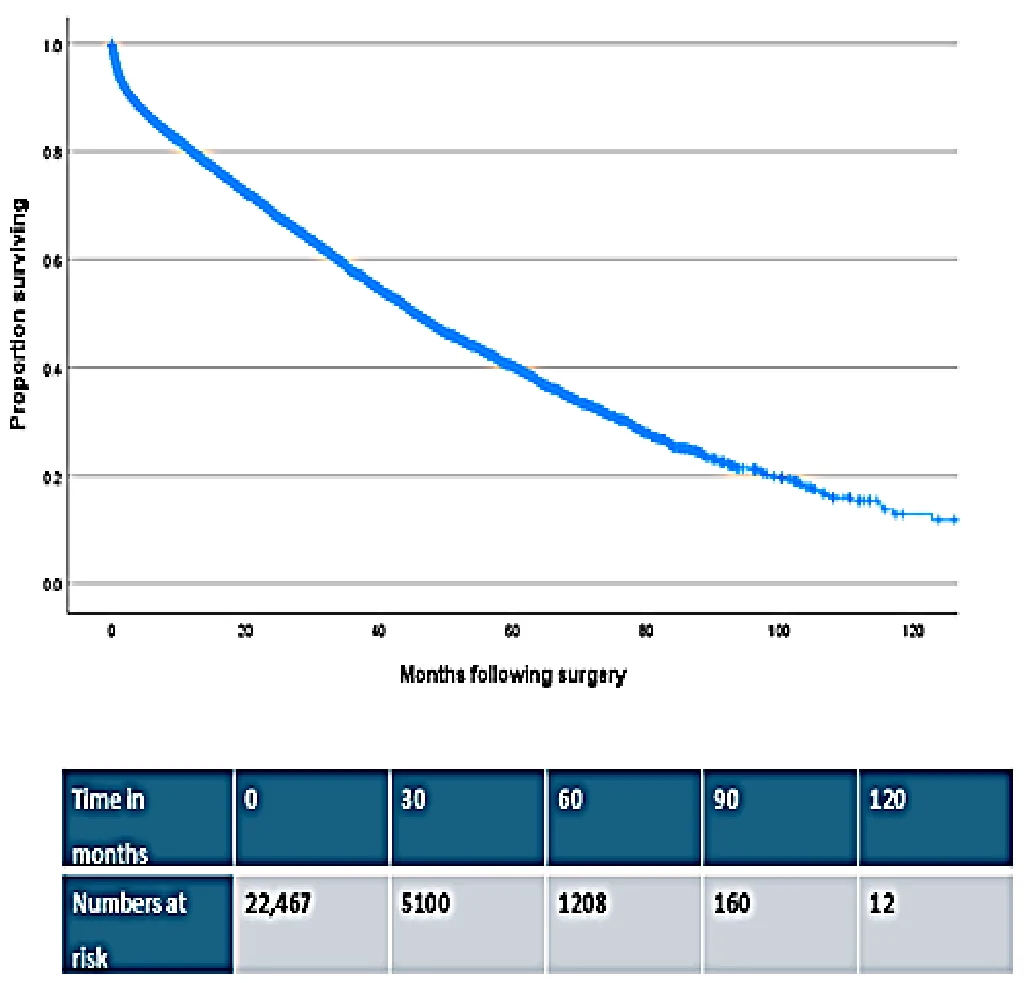
Overall survival. Kaplan–Meier analysis of overall survival following surgery. Kaplan–Meier curve demonstrating overall survival after surgery in the study cohort. The *y*-axis represents the proportion of patients surviving, and the *x*-axis represents time since surgery in months. Survival declined progressively throughout the follow-up period, with an estimated survival probability of approximately 60% at 36 months, 40% at 60 months, and 20% at 100 months. The number of patients at risk at selected time points is shown below the plot (0 months: 22,467; 30 months: 5100; 60 months: 1208; 90 months: 160; 120 months: 12). These findings demonstrate a gradual reduction in long-term survival following surgery over the 10-year follow-up period. The blue line represents the survival over time.

In the follow-up period of 19.5±19.8 months, 7380 patients 32.8% died. Actuarial survival was 40.2 ± 0.6% at 5 years and 12.8 ± 1.8% at 10 years, with a median survival of 45.5 months [Figure 3].

#### 3.4.1. Impact of Concomitant Cardiac Surgery

**Figure 4:**
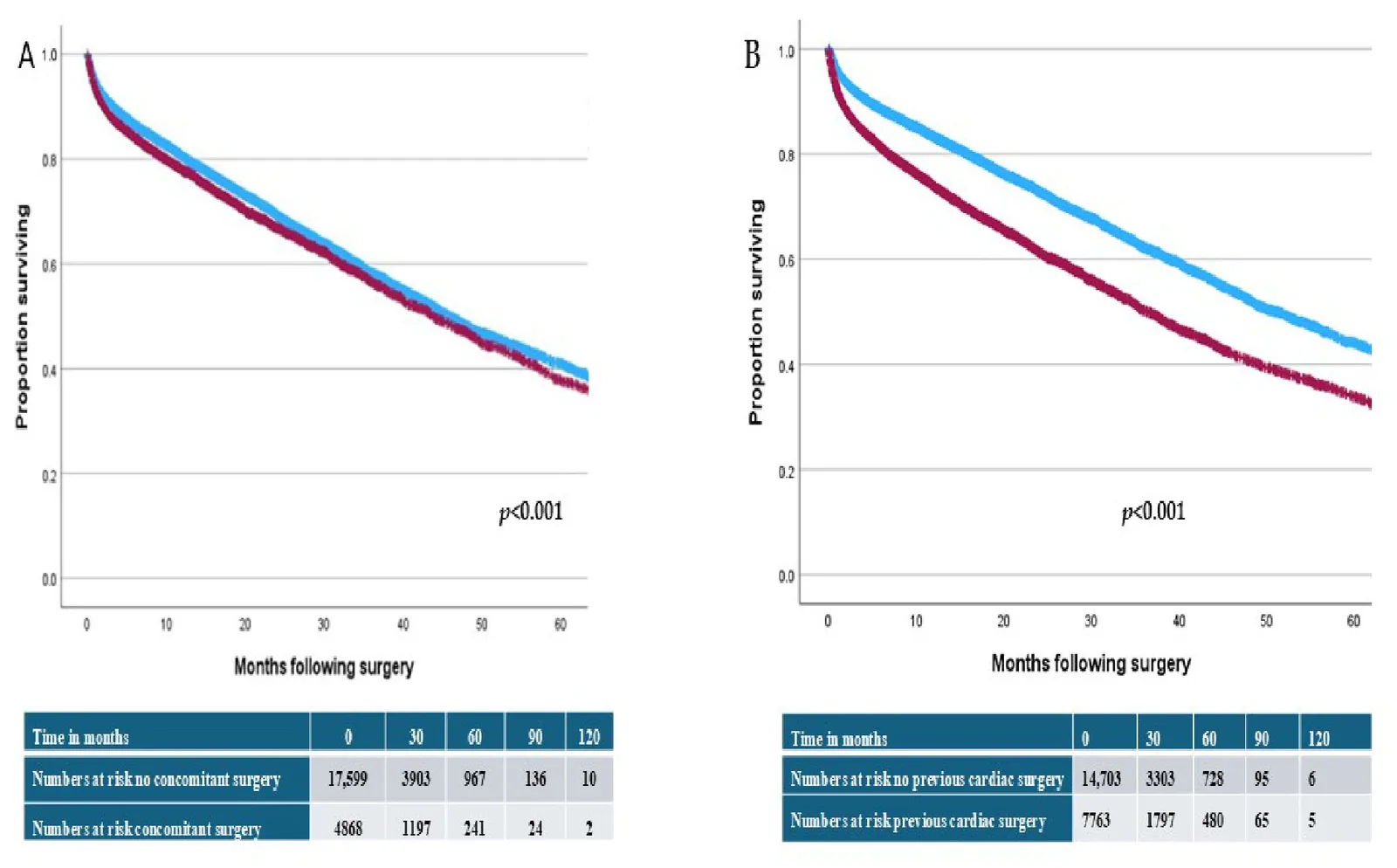
Impact of concomitant cardiac surgery and prior cardiac surgery status on long-term survival. (**A**) Kaplan–Meier curves comparing overall survival among patients who underwent concomitant surgical procedures and those who did not. Patients undergoing concomitant surgery (red line) demonstrated significantly lower long-term survival compared with patients without concomitant surgery (blue line) (log-rank *p* < 0.001). Numbers at risk are presented below the survival curve. (**B**) Kaplan–Meier curves comparing overall survival among patients with and without a history of prior cardiac surgery. Patients with previous cardiac surgery (red line) had significantly reduced survival following the index operation compared with those without prior cardiac surgery (blue line) (log-rank *p* < 0.001). Tick marks indicate censored observations. The corresponding numbers at risk at selected follow-up time points are shown beneath each panel.

Concomitant cardiac surgery at the time of LVAD implantation was associated with reduced long-term survival. Five-year survival was 40.9 ± 0.7 percent and 10-year survival was 13.1 ± 2 percent in patients without concomitant surgery, compared with 37.6 ± 1.3 percent and 12.1 ± 3.3 percent, respectively, in those undergoing concomitant procedures (*p* < 0.001) [Figure 4A].

#### 3.4.2. Impact of Prior Cardiac Surgery

Prior cardiac surgery was associated with reduced long-term survival after LVAD implantation. Five-year survival was 44.1 ± 0.8 percent and 10-year survival was 14.2 ± 2.8 percent among patients without previous cardiac surgery, compared with 33.8 ± 0.9 percent and 10.4 ± 2.1 percent, respectively, among those with a history of cardiac surgery (*p* < 0.001) [Figure 4B].

#### 3.4.3. Univariate Predictors of Long-Term Mortality

Significant univariate preoperative variables included age, previous cardiac surgery, LVAD exchange, and transition from ECMO to durable LVAD (Table 5A). Intraoperatively the significant variables were concomitant cardiac surgery, LVAD with temporary RVAD support, and presence of total artificial heart. Postoperatively significant variables included reoperation for bleeding (<48 and >48 h), ECMO support, presence of IABP, prolonged ventilation, GI bleed, stroke and long-term hemodialysis.

#### 3.4.4. Multivariate Predictors of Long-Term Mortality

Variables significant on univariate analysis were entered into a multivariable Cox regression model (Table 5B). Significant preoperative variables in the multivariate analysis long-term mortality included older age, LVAD exchange, preoperative ECMO support, and prior cardiac surgery. Unsurprisingly, LVAD exchange was associated with a greater HR for long-term mortality within the category of prior cardiac surgery [HR = 1.986 versus HR = 1.246]. Concomitant cardiac surgery was the key intraoperative variable, while postoperative significant variables included stroke, RVAD support, and long-term dialysis (Table 5B).

### 3.5. Development of a Cox Regression Model for Long-Term Risk Stratification for Survival

Using the significant covariates identified in the multivariable Cox regression analysis (age, previous cardiac surgery, female sex, planned concomitant RVAD, preoperative ECMO, concomitant surgery, presence of IABP, stroke (CVA) and postoperative dialysis) (Table 4B), a prognostic risk score was derived from the model’s linear predictor (Table 5C).

The score can be calculated using the formula:

Score = 0.022 × Age + 0.222 × Prev Cardiac Surgery + 0.083 × Sex + 0.812 × Planned Conc RVAD + 0.3 × Pre ECMO + 0.063 × Con Surgery + 0.1 × IABP + 0.594 × CVA + 0.97 × Post Dial (1)

The score incorporates age, concomitant surgery, female sex, prior cardiac surgery, preoperative ECMO, intra-aortic balloon pump (IABP) use, stroke (CVA (cerebrovascular accident)), planned concomitant right ventricular (RV) support and postoperative dialysis with weighted coefficients corresponding to their relative contribution to mortality risk. This model provides an individualized estimate of risk, with higher scores indicating a greater likelihood of adverse outcomes. By integrating readily available clinical and perioperative variables, the scoring system offers a practical and quantitative tool for risk stratification, patient counseling, and clinical decision-making. Further validation may help establish its utility across broader patient populations and clinical settings.

#### 3.5.1. Median Survival by Groups

**Figure 5:**
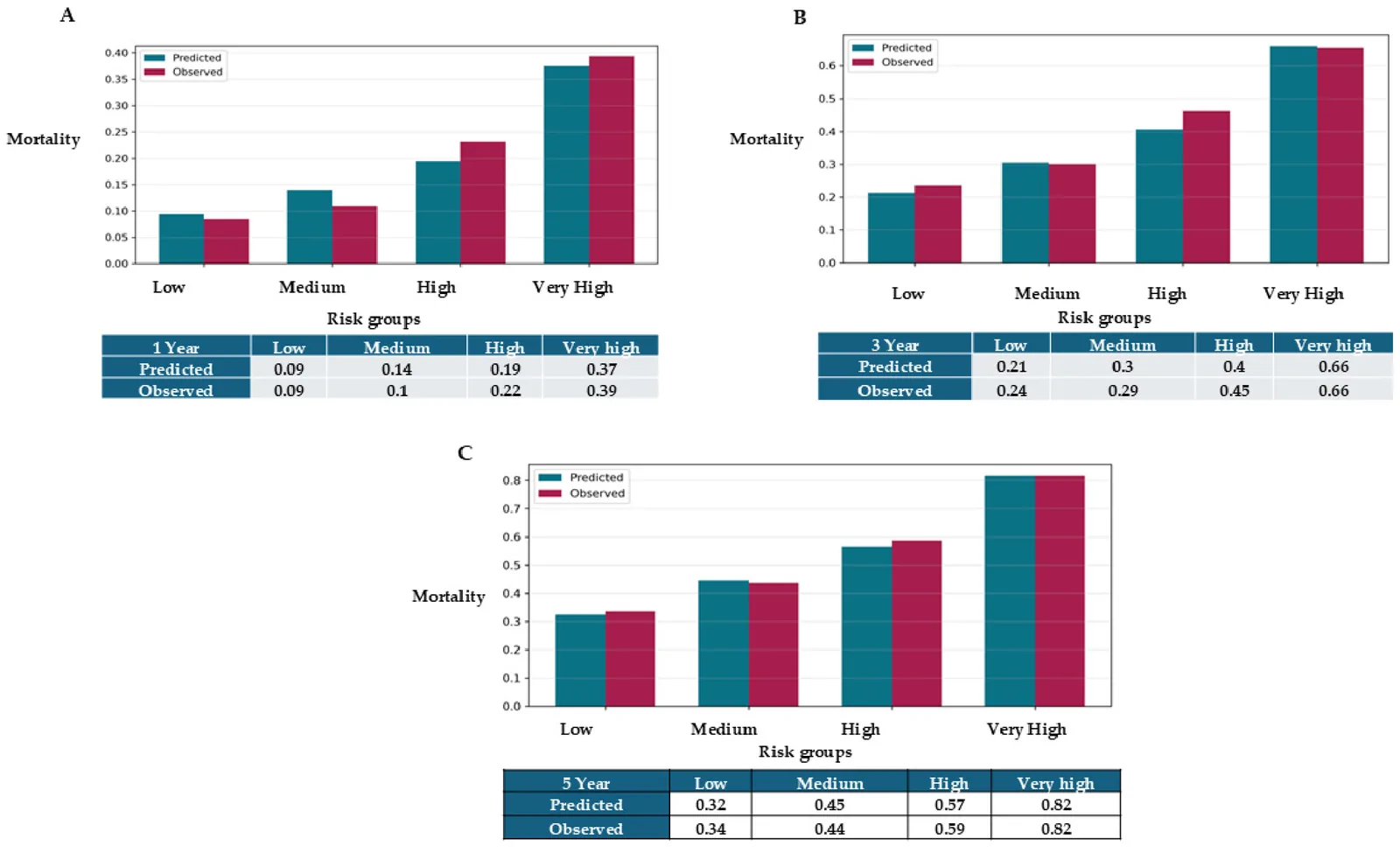
Performance of long-term prediction model. Performance of the long-term mortality prediction model in the test set. (**A**) Comparison of predicted and observed 1-year mortality rates across four risk categories (low, medium, high, and very high) in the test set. Predicted mortality rates were 0.09, 0.14, 0.19, and 0.37, respectively, compared with observed mortality rates of 0.09, 0.10, 0.22, and 0.39. (**B**) Comparison of predicted and observed 3-year mortality rates across risk categories. Predicted mortality rates were 0.21, 0.30, 0.40, and 0.66, compared with observed rates of 0.24, 0.29, 0.45, and 0.66. (**C**) Comparison of predicted and observed 5-year mortality rates across risk categories. Predicted mortality rates were 0.32, 0.45, 0.57, and 0.82, while observed mortality rates were 0.34, 0.44, 0.59, and 0.82. Across all time points, the model demonstrated good calibration, with predicted mortality closely corresponding to observed mortality and maintaining appropriate risk stratification across increasing risk groups.

Patients were stratified into risk categories according to the magnitude of the calculated score, namely low (score ≤ 1.3), medium (1.3 < score ≤ 1.6), high (1.6 < score ≤ 2.1), and very high (score > 2.1). This stratification framework enabled the classification of patients into clinically meaningful prognostic groups based on the estimated hazard derived from the multivariable Cox model. Median survival in the different risk groups was low: 7.5 years, medium: 5.5 years, high: 3.7 years, very high: 1.7 years. The performance of this model at 1, 3 and 5 years is shown in Figure 5A, Figure 5B and Figure 5C, respectively. The final Cox risk score (Table 5C) was constructed by selecting all variables that were significant in the multivariable model (Table 5B), applying clinical parsimony to maintain interpretability, removing collinear predictors, and retaining covariates with strong effect sizes and/or well-established prognostic relevance. Hence the final predictors were chosen on their prognostic utility and interpretability.

#### 3.5.2. Derivation of Kaplan–Meier Curves

**Figure 6:**
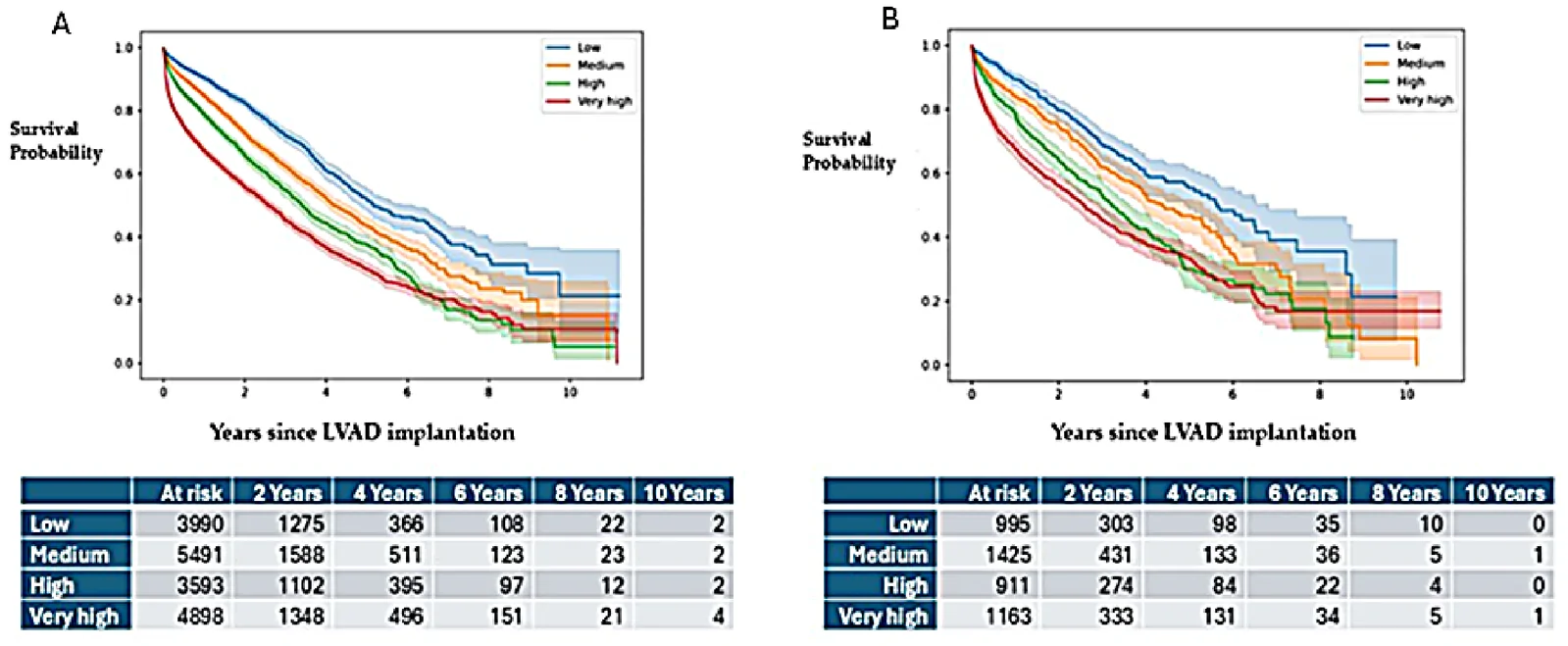
Kaplan–Meier survival curves in training and test sets. Kaplan–Meier survival curves stratified by long-term mortality risk groups in the training and test cohorts. Kaplan–Meier survival analyses demonstrating overall survival according to model-derived risk categories (low, medium, high, and very high risk) following LVAD implantation. (**A**) Training cohort and (**B**) test cohort. Patients classified in progressively higher risk groups experienced correspondingly lower survival probabilities throughout follow-up, with the very high-risk group exhibiting the poorest long-term outcomes and the low-risk group demonstrating the best survival. The survival curves showed clear separation across all risk strata in both cohorts, indicating effective discrimination of long-term mortality risk by the prediction model. Shaded areas represent 95% confidence intervals. Differences in survival among risk groups were statistically significant in both the training and test cohorts (log-rank *p* < 0.001). Numbers at risk at selected follow-up time points are provided below each panel. Abbreviations: LVAD, left ventricular assist device.

Figure 6 demonstrates the ability of the Cox-derived risk score to effectively stratify patients according to long-term survival following LVAD implantation. In both the training and testing cohorts, Kaplan–Meier survival curves showed clear and progressive separation across the low-, medium-, high-, and very high-risk groups, with the low-risk group consistently exhibiting the highest survival probabilities and the very high-risk group experiencing the poorest outcomes. Importantly, this pattern was preserved in the independent test cohort, supporting the reproducibility and generalizability of the prognostic model. The early and sustained divergence of the survival curves suggests that the identified clinical variables capture meaningful differences in baseline risk that continue to influence outcomes throughout long-term follow-up. Although some convergence of the curves is observed at later time points, likely reflecting the smaller number of patients remaining at risk, the overall gradient in survival remains evident. These findings indicate that the risk score provides clinically meaningful discrimination of long-term mortality risk and may serve as a useful tool for preoperative risk assessment, patient counseling, and identification of patients who may benefit from closer surveillance and tailored management strategies after LVAD implantation. The Kaplan–Meier survival curves are shown in Figure 6A,B by Cox risk groups for the training and test sets respectively.

#### 3.5.3. Decision Analysis Curves for Determination of Clinical Utility of 90-Day Model and the Long-Term Cox Model

**Figure 7:**
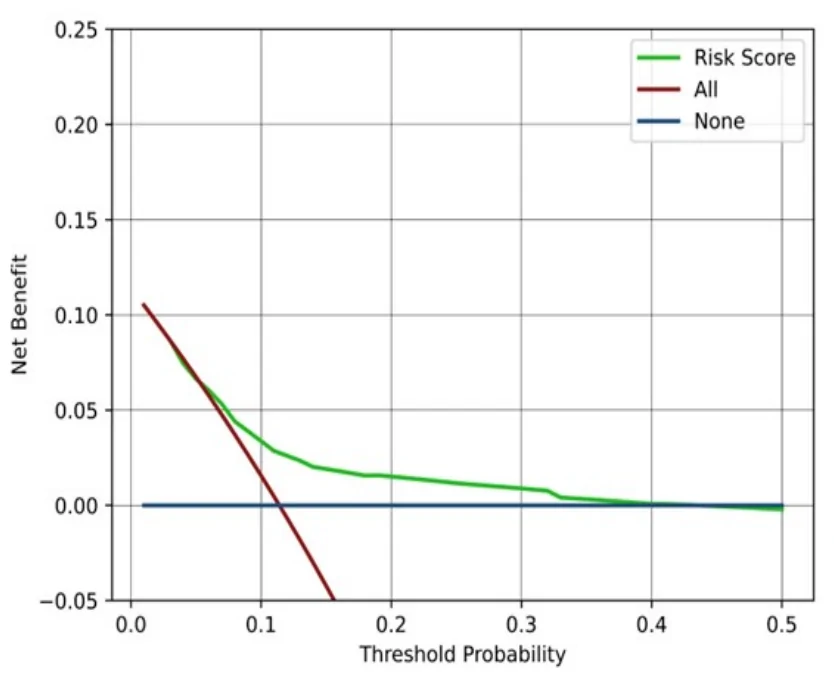
Decision curve analysis of the 90-day mortality risk score. Decision curve analysis (DCA) evaluating the clinical utility of the 90-day mortality risk score across a range of threshold probabilities. The green line represents the net benefit of the risk prediction model, the red line represents a strategy of treating all patients as high risk, and the blue line represents a strategy of treating no patients as high risk.

Decision curve analysis (Figure 7) showed that the 90-day risk score provided greater net benefit than both treat-all and treat-none strategies across threshold probabilities from 5% to 40%. The risk score demonstrated a positive net benefit across a clinically relevant range of threshold probabilities and outperformed both the “treat-all” and “treat-none” strategies, indicating potential value for clinical decision-making and risk stratification. Net benefit gradually declined at higher threshold probabilities but remained favorable over much of the evaluated range, supporting the model’s usefulness for identifying patients at increased risk of 90-day mortality.

**Figure 8:**
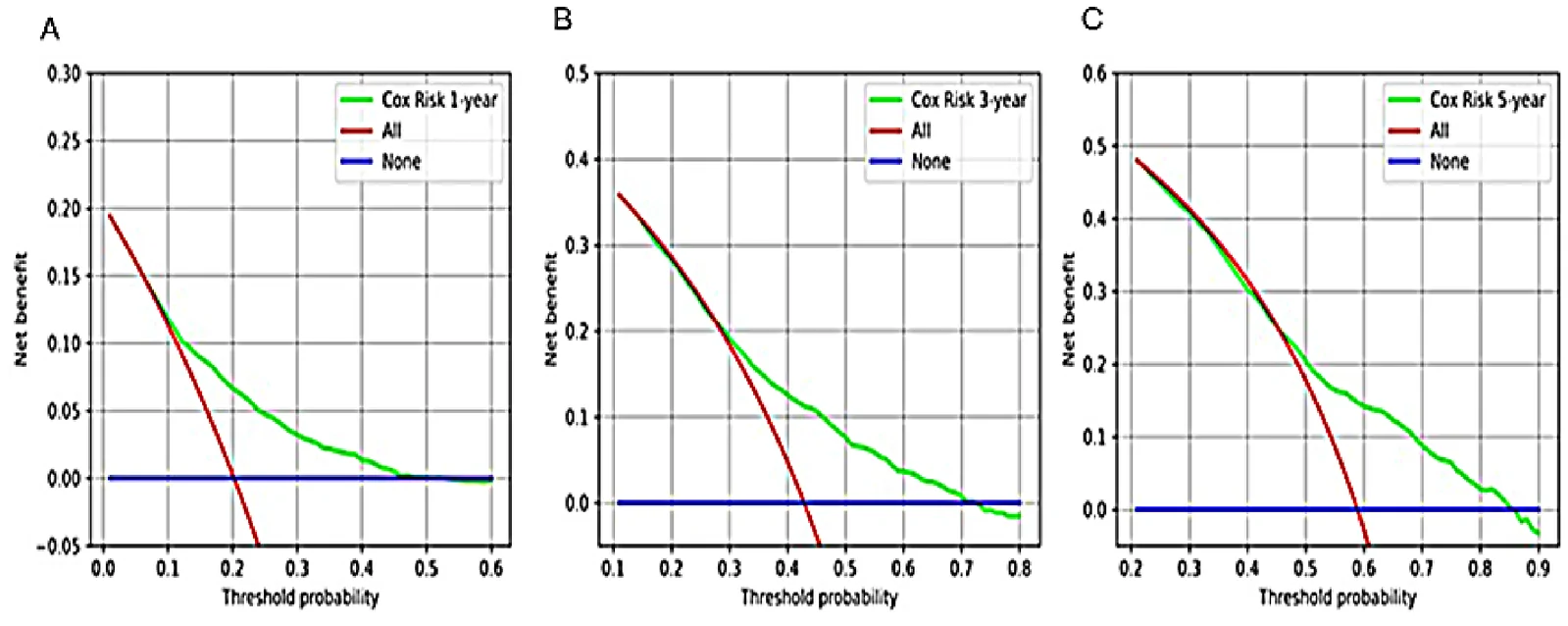
Decision curve analysis of the long-term mortality prediction models at 1, 3, and 5 years. Decision curve analysis (DCA) assessing the clinical utility of the long-term mortality prediction models for (**A**) 1-year, (**B**) 3-year, and (**C**) 5-year mortality. The green line represents the net benefit of the Cox regression-based risk prediction model, the red line represents a strategy of treating all patients as high risk, and the blue line represents a strategy of treating no patients as high risk.

Figure 8 shows decision curve analysis that demonstrates the clinical utility of the Cox regression model. Decision curve analysis demonstrated that the Cox model provides clinical value by yielding a higher net benefit than both treat-all and treat-none strategies across a range of clinically relevant risk thresholds. These findings suggest that incorporating model-based risk estimates into clinical decision-making may improve the balance between identifying high-risk patients and avoiding unnecessary interventions. The Cox risk model provided a greater net benefit than treat-all and treat-none strategies across threshold probabilities of 10–50% at 1 year, 30–70% at 3 years, and 45–85% at 5 years, suggesting potential clinical utility within these range. Across a broad range of threshold probabilities, the prediction models demonstrated greater net benefit than both the treat-all and treat-none strategies, indicating potential clinical value for individualized risk stratification and decision-making. Net benefit was maintained over clinically relevant thresholds for each prediction horizon, supporting the utility of the model in identifying patients at increased risk of long-term mortality following LVAD implantation. Abbreviations: DCA, decision curve analysis; LVAD, left ventricular assist device.

#### 3.5.4. Effect of Propensity Matching on the 90-Day and Long-Term Mortality Models

Age and sex were included in the propensity score model. Following 1:1 propensity score matching, all 4868 patients who underwent concomitant surgery were successfully matched to 4868 patients without concomitant surgery, providing a matched cohort of 9736 patients. Before matching, age showed a modest imbalance (absolute SMD = 0.082), which was fully resolved post-matching (absolute SMD = 0.0005). Sex exhibited minimal baseline imbalance (absolute SMD = 0.032) and was similarly reduced to near zero following matching (absolute SMD = 0.001). Covariate balance was substantially improved after matching, with both covariates achieving absolute SMDs < 0.001.

The association was substantially stronger during the early post-implant period. Within 90 days, the Kaplan–Meier survival curves differed significantly between the two groups (log-rank *p* < 0.001), and concomitant surgery was associated with a higher hazard of death in the matched cohort (HR 1.61, 95% CI 1.41–1.83). Although concomitant surgery was associated with increased mortality over the entire follow-up period, the magnitude of this association was greater during the first 90 days after implantation and was attenuated during longer-term follow-up.

**Figure 9:**
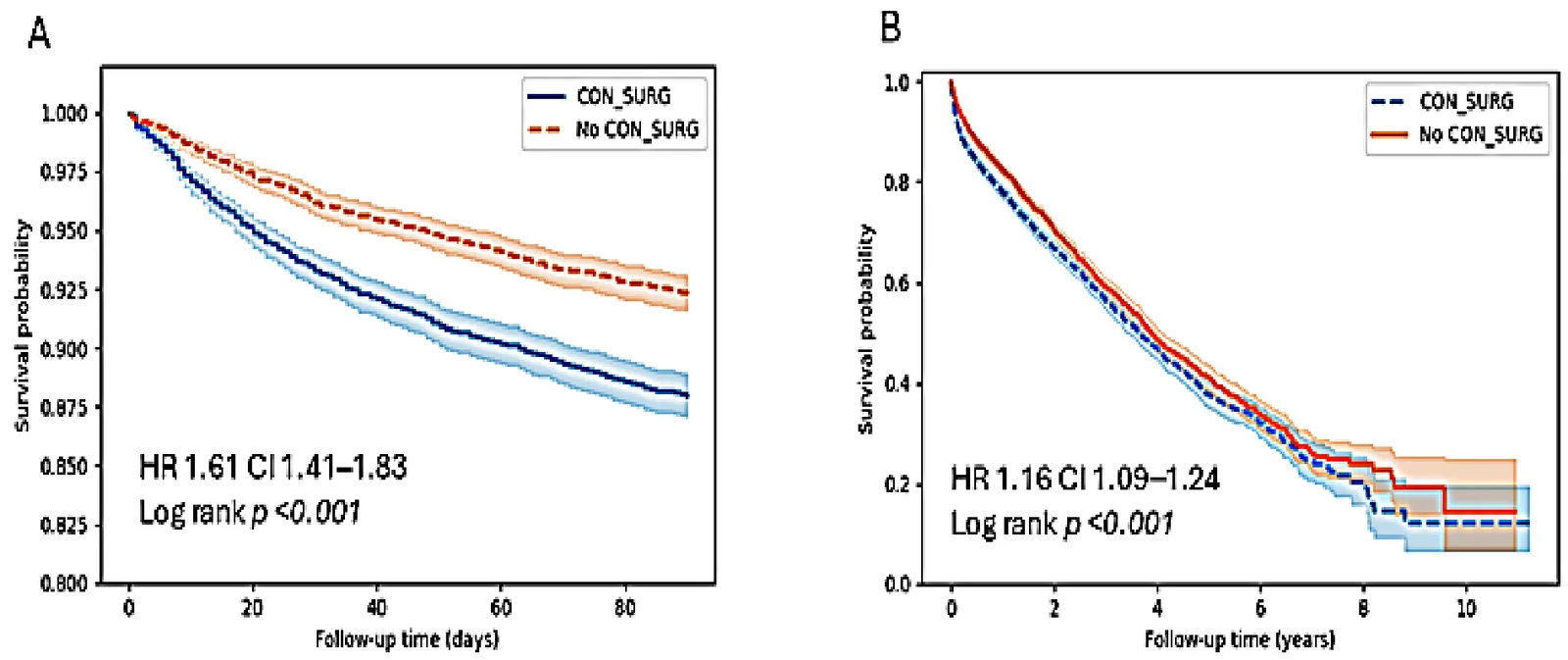
Kaplan–Meier survival curves comparing outcomes between the concomitant cardiac surgery and non-concomitant cardiac groups after propensity score matching. (**A**) In the 90-day risk model, patients in the concomitant cardiac surgery group ([HR] 1.61, 95% confidence interval [CI] 1.41–1.83; log-rank *p* < 0.001)). (**B**) In the long-term Cox regression model, the concomitant cardiac surgery group also demonstrated an increased risk of mortality ([HR] 1.16, (95% CI 1.09–1.24); log-rank *p* < 0.001)). The shaded areas represent the confidence intervals.

Patients who underwent concomitant surgery demonstrated slightly poorer survival over the follow-up period compared with those who did not. Median survival was 3.65 years among patients who underwent concomitant surgery and 3.89 years among those without concomitant surgery. The Kaplan–Meier survival curves differed significantly between the two matched groups (log-rank *p* < 0.001). Consistent with this finding, Cox proportional hazards analysis of the matched cohort showed that concomitant surgery was associated with a higher overall hazard of death (HR 1.16, 95% CI 1.09–1.24) in Figure 9.

## 4. Discussion

### 4.1. Factors That Impact Survival Post LVAD Implantation

Despite remarkable advances in LVAD technology and patient management over the past two decades, outcomes remain strongly influenced by a range of patient-related, procedural, and post-implantation factors. Patients requiring additional surgical interventions, mechanical circulatory support, or those who develop major complications after LVAD implantation often represent a particularly high-risk population with significantly worse short- and long-term survival. Conditions such as right ventricular failure, the need for RVAD or BiVAD support, redo cardiac surgery, pump exchange, concomitant cardiac procedures, ECMO bridging, gastrointestinal bleeding, stroke, peripheral vascular disease, and renal failure all reflect the complex interplay between advanced heart failure, comorbid disease burden, and the challenges of long-term mechanical support. Understanding the impact of these factors is essential for risk stratification, patient selection, perioperative planning, and the optimization of outcomes following LVAD implantation.

#### 4.1.1. Right Ventricular Failure

Right ventricular failure affects 10–40% of LVAD recipients, with about 10% requiring temporary RVAD support, and is consistently linked to worse perioperative and long-term survival compared with isolated LVAD support [3,4,7,8,9]. In our cohort, both RVAD implantation and BiVAD support were associated with significantly poorer 90-day and long-term outcomes.

Kormos et al. reported RV failure rates at approximately 20% after HeartMate II implantation with markedly reduced survival among RVAD-dependent patients, while Fitzpatrick et al. showed that even planned BiVAD implantation though superior to delayed conversion remained inferior to isolated LVAD support, a pattern consistently reproduced in larger INTERMACS cohorts [3,9,10].

#### 4.1.2. Redo Cardiac Surgery

Our multivariate analysis found that patients undergoing LVAD implantation after prior cardiac surgery had a 25% lower 5-year survival, with a hazard ratio of 1.28 indicating worse outcomes compared with those without previous surgery.

Current literature remains heterogenous. Ayers et al., in a cohort of 321 patients, reported that redo cardiac surgery at LVAD implantation increased operative complexity and showed a nonsignificant trend toward reduced survival, likely reflecting limited statistical power [11]. In contrast, INTERMACS analyses of more than 25,000 patients demonstrated significantly higher mortality among those with prior cardiac surgery (HR 1.57) and an even greater risk in patients undergoing concomitant procedures (HR 1.73) [10].

#### 4.1.3. Pump Exchange

LVAD exchange carries high mortality. In a 152-patient series, increasing prior sternotomies strongly predicted mortality (HR 3.1–5.2) and 1-year survival was ~48%, supporting non-sternotomy approaches for better perioperative outcomes [12,13,14]. A 17-patient HeartMate II series similarly showed superior operative and long-term results with subcostal access [15]. Consistent with these findings, our INTERMACS analysis showed increased perioperative (OR 6.9) and late mortality (HR 1.26), and institutional outcomes demonstrated 1- and 5-year survival of 72.5 ± 6.5% and 39 ± 8%, with non-sternotomy approaches yielding fewer complications and improved long-term survival [16].

#### 4.1.4. Concomitant Surgery

Earlier studies linked cardioplegic arrest to increased mortality in patients with ventricular dysfunction, though more recent data show mixed effects during LVAD implantation [17,18]. Sugiura et al. found no significant mortality difference with concomitant valve surgery in a small series [19]. Kawabori et al. observed higher RVAD use and lower short- and long-term survival with cardioplegia, though these differences were not statistically significant [20]. EUROMACS data showed longer ICU stays, more bleeding, and worse long-term survival with cardioplegic arrest during left-sided valve surgery, whereas non-cardioplegic procedures such as tricuspid repair or ASD closure were not associated with adverse outcomes and may even protect against late RV failure [21,22]. In our analysis, concomitant surgery particularly when cardioplegia was inferred was associated with higher early mortality and modestly reduced long-term survival consistent with prior INTERMACS findings [23].

Concomitant surgery without cardioplegia was linked to higher 90-day mortality (OR 1.19), with an even greater risk for procedures requiring cardioplegia (OR 1.33), aligning with INTERMACS data showing that concomitant cardiac surgery predicts increased long-term mortality (HR 1.73) [10].

#### 4.1.5. Preoperative ECMO Support

Several studies have evaluated outcomes in INTERMACS 1 patients bridged to LVAD with ECMO, with mixed results. In a series of LVAD exchanges, Contreras reported poor long-term survival among ECMO-supported patients [11]. Unai et al. have shown comparable short- and mid-term outcomes between ECMO-bridged and directly implanted patients [24]. Han et al. and Lamba et al. showed recently that ECMO stabilization may improve hemodynamics and end-organ function without adversely affecting 30-day or 1-year survival, although sample sizes remain limited [25,26].

Pagani et al. showed that ECMO can successfully bridge critically ill patients to LVAD implantation when end-organ function recovers, yielding survival comparable to direct implantation [27]. Our cohort of 1009 INTERMACS 1 patients bridged with ECMO carried substantially higher perioperative risk, including increased 90-day mortality (OR 1.85) and reduced 5-year survival (HR 1.23), likely due to both profound shock severity and ECMO-related complications. These results are consistent with prior INTERMACS analyses demonstrating that critical shock at implantation markedly increases early postoperative mortality (HR 1.58) [10,28].

#### 4.1.6. GI Bleeding

Magnetically levitated centrifugal LVADs show markedly better outcomes than axial-flow pumps, with the 14th STS INTERMACS report (29,391 patients, 2013–2022) demonstrating fewer hemocompatibility complications including higher 5-year freedom from GI bleeding (72% vs. 60%) and superior survival at both 1 year (86% vs. 79%) and 5 years (64% vs. 44%) [29].

#### 4.1.7. Stroke

Magnetically levitated centrifugal LVADs showed significantly higher freedom from stroke at 1 year (93% vs. 83%) and 5 years (87% vs. 67%) compared with older devices [29]. In our cohort, stroke was a major predictor of long-term mortality (HR 1.81), and patients who experienced a stroke had markedly lower survival (21.1% vs. 46.4%).

#### 4.1.8. Peripheral Vascular Disease

Peripheral arterial disease (PAD) is a strong predictor of adverse outcomes after LVAD implantation. In a propensity-matched National Inpatient Sample analysis, PAD was associated with a 1.7-fold increase in adjusted in-hospital mortality [30]. Consistent with this, INTERMACS data demonstrated significant impact on long-term survival in PAD patients (HR 2.23) [10].

#### 4.1.9. Renal Failure

Chronic hemodialysis creates large fluid shifts that destabilize LVAD physiology, with dialysis-associated volume depletion leading to hypotension and reduced flows, and volume overload in between dialysis runs contributing to right-sided dysfunction. These fluctuations are associated with frequent hospitalizations and poorer long-term outcomes, with only 11% of magnetically levitated centrifugal LVAD-supported patients remaining free from readmission at 5 years [29]. Renal dysfunction affects 30–45% of patients with heart failure, and post-LVAD renal failure increases the risk of bleeding, infection, stroke, and RV failure [31]. In our cohort, long-term hemodialysis was associated with markedly reduced 5-year survival (18.2% vs. 47.4%; HR 2.82). Although renal function improves in approximately 60% of patients after LVAD implantation, persistent dialysis dependence carries a 6-month mortality of up to 75% and is considered a contraindication for destination LVAD therapy by the International Society for Heart and Lung Transplantation [32].

### 4.2. Development of a Risk Scoring System

Prior studies developed LVAD risk scores using variables such as serum albumin, bilirubin, ventricular dimensions, and hemodynamics, but these models demonstrated limited clinical utility [33]. Subsequent scoring systems incorporating age, prior cardiac surgery, renal function, and CVP-to-PCWP ratios improved risk stratification [34]. More recent efforts have applied machine learning to predict right ventricular failure [35,36]. However, none have provided an individualized estimate of postoperative mortality that can be used during informed consent. We analyzed INTERMACS data from over 22,400 durable LVAD recipients, including axial-flow (HeartMate II) and magnetically levitated devices (HeartWare International, Inc., Framingham, MA, USA; Heart Mate 3, Abbott, Pleasanton, CA, USA), to identify complications predictive of short- and long-term mortality.

We therefore developed a risk model incorporating preoperative variables to estimate 90-day mortality as a percentage. To our knowledge, this is the first model designed to provide quantitative post-implantation mortality risk, and despite limitations inherent to INTERMACS variable availability, these estimates may aid in counseling and decision-making for patients undergoing high-risk LVAD surgery. This study also includes a long-term survival model using Cox regression analysis enabling stratification of patients into clinically meaningful risk groups.

#### 4.2.1. Clinical Utility the 90-Day Mortality Score

The FasterRisk-derived 90-day mortality score identified seven clinically meaningful predictors of adverse outcomes following mechanical circulatory support. Device type emerged as the strongest determinant of risk, followed by advanced age and preoperative ECMO support. Prior cardiac surgery and concomitant RVAD support were also retained in the final model, suggesting that procedural complexity and underlying right ventricular dysfunction contribute independently to early mortality. Unlike traditional regression-based risk models, FasterRisk generated a sparse, integer-weighted scoring system that preserves interpretability while enabling rapid bedside risk assessment. The score stratified patients across a broad risk spectrum, with estimated 90-day mortality ranging from 3.6% to 75%, supporting individualized patient counseling, clinical decision-making, and risk-adjusted outcome assessment. Importantly, compared with established LVAD risk models such as the HeartMate II Risk Score and EUROMACS-based models, this score relies on a smaller number of readily available clinical variables while maintaining transparency regarding the relative contribution of each predictor. By combining machine learning–based variable selection with the simplicity of a point-based bedside tool, the model offers a practical approach for preoperative risk stratification and identification of patients at heightened risk of early mortality [37,38].

The broad risk gradient observed across score categories supports its potential use in patient counseling, shared decision-making, resource allocation, and risk-adjusted outcome assessment. Furthermore, its transparent integer-based structure makes it particularly suitable for implementation within electronic health records and routine clinical workflows.

#### 4.2.2. Decision Curve Analysis for 90-Day Risk Model

Decision curve analysis demonstrated that the 90-day risk score provided greater net benefit than both treat-all and treat-none strategies across threshold probabilities ranging from 5% to 40%. These findings suggest that the model may offer meaningful clinical utility by supporting risk-guided decision-making within this range. Specifically, use of the risk score could improve identification of patients at elevated risk of the outcome while minimizing unnecessary interventions among lower-risk individuals. The broad range of threshold probabilities over which the model showed superior net benefit indicates potential applicability across diverse clinical scenarios and risk tolerances. Importantly, these results complement traditional measures of model performance by demonstrating not only predictive accuracy but also the potential clinical value of incorporating the risk score into patient management. Taken together, the decision curve analysis supports the use of the 90-day risk score as a tool to enhance individualized risk stratification and optimize clinical decision-making. The model demonstrated moderate discrimination (C-statistic = 0.69) and provided greater net benefit than treat-all and treat-none strategies for threshold probabilities between 5% and 40%. The absence of net benefit outside this range may reflect limited discrimination at the extremes of risk, where classification errors have greater clinical consequences. Future work incorporating additional prognostic variables, nonlinear modeling approaches, and recalibration strategies may improve performance across a broader range of decision thresholds.

#### 4.2.3. Clinical Utility of the Long-Term Survival

The Cox regression model highlights the long-term risk factors. This score can help tailor the intensity and frequency of follow-up care after LVAD implantation by identifying patients at different levels of long-term mortality risk. Low-risk patients may be suitable for standard follow-up schedules and routine surveillance. Intermediate-risk patients may benefit from more frequent outpatient assessments, particularly during the first few years after implantation. High- and very high-risk patients may require closer monitoring for right ventricular dysfunction, end-organ complications, device-related events, and recurrent hospitalizations. These patients may also benefit from earlier multidisciplinary interventions involving heart failure, LVAD, and palliative care teams.

#### 4.2.4. Decision Curve Analysis of the Long-Term Cox Model

Decision curve analysis demonstrated the potential clinical utility of the Cox regression model by showing a greater net benefit than both the treat-all and treat-none strategies across a broad range of clinically relevant threshold probabilities. These findings indicate that use of the model to guide clinical decision-making may result in a more favorable balance between identifying patients at increased risk and avoiding unnecessary interventions in lower-risk individuals.

The widening of the threshold ranges associated with clinical utility over longer prediction horizons suggests that the model may provide value particularly in informing intermediate and long-term risk assessment. Importantly, decision curve analysis evaluates the clinical consequences of model-guided decisions and therefore complements traditional measures of model performance, such as discrimination and calibration. The observed net benefit across multiple time points supports the notion that the Cox model is not only statistically robust but also clinically actionable.

Collectively, these results suggest that incorporating the Cox risk model into routine clinical practice may enhance individualized risk stratification and support more informed treatment decisions. Future studies evaluating the model in external populations and real-world clinical settings are warranted to further establish its impact on patient outcomes and healthcare resource utilization.

#### 4.2.5. Impact of Propensity Matching on the 90-Day and Long-Term Mortality Risk Models

Propensity score matching was not part of the primary modeling strategy because the principal objective of this study was risk prediction rather than estimation of a causal treatment effect. Concomitant surgery was retained in the multivariable Cox model because it provided independent prognostic information for survival after adjustment for other candidate predictors. Propensity score matching was subsequently performed as a sensitivity analysis to assess whether the observed association might be attributable to differences in baseline characteristics between patients with and without concomitant surgery. This analysis demonstrated that the association between concomitant surgery and mortality remained after matching, but its magnitude varied over time, with the strong association observed during the first 90 days after implantation and a smaller association over the full follow-up period. These findings support the robustness of the association between concomitant cardiac surgery status and adverse survival outcomes, even after accounting for potential confounding factors through propensity score matching.

#### 4.2.6. Postoperative Stroke and Dialysis

Although postoperative stroke and dialysis emerged as strong predictors of long-term mortality, these events occur after LVAD implantation and therefore are not appropriate for use in preoperative counseling. Accordingly, they were excluded from the preoperative risk model and are applied only in the context of long-term prognostication. Their inclusion in the long-term survival model reflects their substantial impact on long-term outcomes. Hence, stroke and post dialysis are risk factors that are not intended for use in guiding “preoperative” decision-making or counseling of patients. The long-term model is primarily a postoperative prognostic tool, whereas preoperative counseling should rely only on the 90-day risk model for mortality.

#### 4.2.7. Comparison of the Long-Term Risk Score Presented in This Study Versus the Heart Mate Risk Score (HMRS) and the HeartMate 3 Risk Score (HM3RS)

The predictive performance of the present risk model for long-term mortality is comparable to that reported for both the HeartMate Risk Score (HMRS) and the HeartMate 3 Risk Score (HM3RS). In the current study, the model achieved c-statistics of 0.69 in the training cohort and 0.67 in the testing cohort, demonstrating moderate discrimination and consistent performance across cohorts. External validation studies of the HMRS have reported AUC values of approximately 0.70 for early mortality prediction after LVAD implantation, while real-world evaluations of the HM3RS have demonstrated AUC values ranging from 0.63 to 0.69 for 1-year mortality prediction. Although discrimination is broadly similar across these models, important differences exist in their intended use and clinical applicability.

The HMRS was developed primarily to predict short-term mortality in HeartMate II recipients and relies largely on demographic, laboratory, and center-volume variables [38]. As a result, it may not fully capture perioperative factors that influence long-term outcomes. Similarly, the HM3RS was derived from the MOMENTUM 3 clinical trial population to estimate 1- and 2-year mortality among HeartMate 3 recipients and has shown reduced performance in some real-world and contemporary patient populations [39,40]. In contrast, the present model was specifically designed to stratify long-term mortality risk and incorporates clinically relevant perioperative factors, including preoperative ECMO, concomitant right ventricular support, prior cardiac surgery, and concomitant surgical procedures. These variables reflect both patient acuity and operative complexity, factors that are known to influence survival after LVAD implantation.

Furthermore, the proposed score demonstrated clear separation of Kaplan–Meier survival curves across risk strata in both derivation and validation cohorts, suggesting robust discrimination of long-term outcomes. Therefore, rather than replacing existing scores, this model may complement HMRS and HM3RS by providing a practical tool specifically focused on long-term mortality risk assessment and risk-adapted follow-up after LVAD implantation. External multicenter validation and direct head-to-head comparisons with HMRS and HM3RS are warranted to determine its incremental prognostic value.

### 4.3. Validation of Risk Scores

While the proposed risk score demonstrated consistent discrimination and effective risk stratification in both the training and testing cohorts, additional validation is required before widespread clinical implementation. Future studies should focus on external validation in independent multicenter cohorts, assessment across different LVAD generations and patient populations, and prospective evaluation of its impact on clinical decision-making. Further analyses should also examine model calibration over time, clinical utility using decision curve analysis, and performance in important subgroups such as destination therapy and bridge-to-transplant patients in other databases. To reduce temporal bias, we recommend that future work incorporate implant year, device generation, and perioperative era as core covariates or stratification variables, positioning our current models as a foundation that requires temporal updating rather than a definitive, time-invariant risk tool. This validation would be best achieved in prospective independent cohorts.

## 5. Strengths

This study has several important strengths. First, it utilized a very large, contemporary cohort of 22,467 adult patients from the INTERMACS registry, making it one of the largest analyses of LVAD outcomes and enhancing the statistical power and generalizability of the findings. The study examined both short-term (90-day) and long-term mortality, providing a comprehensive assessment of outcomes after LVAD implantation. In addition, we employed robust analytical methods, including multivariable logistic regression, LASSO feature selection, Cox proportional hazards modeling, and the FasterRisk machine learning approach. The use of an 80/20 train-test split and five-fold cross-validation helped reduce overfitting and strengthened the internal validation of both prediction models. Furthermore, the resulting risk scores are based on clinically relevant and readily available variables, making them potentially useful for bedside risk stratification, patient counseling, and perioperative decision-making. The clinical utility of the model is further supported by the decision curve analysis, which demonstrated a greater net benefit than both treat-all and treat-none strategies across clinically relevant threshold probabilities.

## 6. Limitations

Despite its strengths, the study has several limitations. The retrospective observational design limits the ability to establish causal relationships between identified risk factors and mortality. As the analysis relied on registry data, it is susceptible to missing data, reporting errors, and unmeasured confounding variables that may influence outcomes. The predictive performance of both models was acceptable (90-day mortality model c-statistic 0.67–0.69; long-term survival model c-index 0.70–0.72), indicating moderate discrimination for individual patient risk prediction. Future model refinement may be achieved through incorporation of additional prognostic markers, including biomarkers, imaging parameters, and longitudinal clinical data. The use of flexible modeling techniques to account for nonlinear associations and interactions, along with recalibration and external validation in larger cohorts, may further enhance predictive accuracy and potentially broaden the range of decision thresholds over which the model provides clinical utility.

The study needs external validation in independent cohorts to augment confidence in the model’s generalizability to different institutions, healthcare systems, and future LVAD populations. Finally, changes in LVAD technology and management practices over the decade-long study period may impact outcomes and introduce a component of temporal bias. Temporal bias is an important limitation because INTERMACS spans major changes in LVAD technology and perioperative practice, including the transition from HeartMate II to HeartMate 3 and evolving management strategies. These era shifts likely influenced both early outcomes through reductions in pump thrombosis and improved perioperative stability and long-term hazards, given improved device durability and fewer adverse events with newer systems. As implant year and device generation were not incorporated, some risk estimates may reflect historical practice patterns rather than true patient-level predictors. Although analysis in HM 3-exclusive single center prospectively collected data would be required to fully address this bias, its impact can be mitigated by interpreting results in terms of relative risk rather than absolute estimates, emphasizing the need for external validation in contemporary HM3-dominant independent cohorts. We recommend that future models explicitly include temporal core covariates to ensure ongoing relevance in prospective cohorts.

## 7. Conclusions

In this large INTERMACS registry retrospective analysis of 22,467 patients undergoing LVAD implantation, advanced age, preoperative ECMO support, prior cardiac surgery, concomitant cardiac surgery, and RV support were identified as important predictors of adverse outcomes. Prior cardiac surgery and concomitant procedures were associated with both increased early mortality and reduced long-term survival.

Using preoperative factors, we developed and internally validated a machine learning–based 90-day mortality score and a Cox regression-based long-term survival model that demonstrated moderate discrimination and calibration in predicting long-term survival.

Interpretability and predictive accuracy were balanced by using FasterRisk to develop the 90-day mortality model and Cox regression to characterize long-term risk. In this large INTERMACS cohort, we identified key determinants of both perioperative and long-term mortality. To our knowledge, this is the first perioperative mortality risk score that integrates preoperative variables with moderate discriminative performance achieved through machine learning methods, complemented by a Cox model for long-term survival estimation. Although INTERMACS provides a limited set of variables, the resulting models yield clinically meaningful risk estimates that may assist in counseling high-risk LVAD candidates. Internal cross-validation supports model robustness, but external validation remains necessary.

These risk assessment tools may aid preoperative counseling, perioperative planning, and long-term follow-up of LVAD recipients, although external validation is needed before routine clinical implementation.

## Figures and Tables

**Table 1:** Baseline characteristics of study cohort.

Baseline Variables	Magnitude
Age	18–80 y
Sex	
Female	21.3%
Male	78.7%
Prior Cardiac Surgery	34.5%
Pulmonary HTN	24.7%
GI Bleeding	21.6%
Concomitant cardiac surgery	21.7%

**Table 2:** Distribution of concomitant cardiac surgery.

Type of Surgery	Number of Patients	Percentage of Total
Ventricular septal defect closure	43	0.2
Mitral valve replacement with bio prosthesis	126	0.5
Mitral valve replacement mechanical	33	0.1
Mitral valve repair with annuloplasty ring	674	3
Coronary artery bypass graft	568	2.6
Aortic valve replacement mechanical	109	0.5
Aortic valve replacement bio prosthesis	476	2.2
Aortic valve repair non-closure	144	0.6
Aortic valve repair closure	205	0.9
Pulmonary valve replacement mechanical	12	0.1
Pulmonary valve replacement with bio prosthesis	9	0.1
Pulmonary valve repair	9	0.1
Tricuspid valve repair with annuloplasty ring	1184	5.3
Tricuspid valve repair DeVega	108	0.5
Tricuspid valve repair other techniques	1714	7.6
Tricuspid valve replacement mechanical	67	0.3
Tricuspid valve replacement with bio prosthesis	116	0.5
Closure of atrial septal defect/patent foramen ovale	4166	19.0

**Table 3:** (**A**) Univariate analysis for 90-day mortality. (**B**) Multivariable analysis for 90-day mortality.

(**A**)				
Variable	Number	Percentage	OR (95% CI)	*p* Value
Preoperative variables				
ECMO	1009	4.5	2.71 (2.3, 3.18)	<0.001
Right atrial pressure			1.01 (1.01, 1.02)	<0.001
Pulmonary capillary wedge pressure			0.99 (0.99, 1)	<0.001
Every year increasing age > 55 years			1.02 (1.02, 1.03)	<0.001
LVAD exchange	622	2.8	3.54 (1.69, 7.42)	<0.001
Intraoperative variables				
Concomitant surgery	4868	21.7	1.27 (1.15, 1.4)	<0.001
Pulmonary hypertension > 3 Wood units			0.88 (0.79, 0.98)	0.018
Temporary RVAD support	1335	5.9	5.07 (4.3, 5.97)	<0.001
Postoperative variables				
Prolonged ventilation > 24 h	303	1.1	2.25 [1.97, 2.58]	<0.001
Delayed RVAD	191	0.9	1.92 (1.3, 2.82)	<0.001
Reoperation for bleeding > 48 h	842	3.7	2.45 (2.05, 2.91)	<0.001
Reoperation for bleeding < 48 h	888	4.0	1.74 (1.44, 2.1)	<0.001
ECMO	785	3.5	3.13 (2.63, 3.72)	<0.001
Gastrointestinal bleed	280	1.0	0.46 (0.4, 0.52)	<0.001
Stroke	444	1.97	1.34 (1.2, 1.5)	<0.001
Dialysis	1102	4.9	5.3 (4.83, 5.81)	<0.001
(**B**)				
	Odd Ratio	95% CI Lower	95% CI Upper	*p* Value
Preoperative variables				
Every year or increasing age > 55 years	1.035	1.031	1.039	<0.001
ECMO support	1.562	1.247	1.956	<0.001
LVAD exchange	3.13	1.339	7.315	0.008
Intraoperative variables				
Concomitant cardiac surgery	1.224	1.097	1.366	<0.001
Temporary RVAD support	5.07	4.3	5.97	<0.001
Postoperative variables				
Prolonged ventilation > 24 h	1.277	1.082	1.507	0.004
ECMO support	1.367	1.06	1.764	0.016
Reoperation for bleeding > 48 h	1.804	1.487	2.188	<0.001
Reoperation for bleeding < 48 h	1.326	1.079	1.63	0.007
Stroke	1.328	1.179	1.496	<0.001
Dialysis	4.52	4.104	4.979	<0.001

ECMO: Extracorporeal membrane oxygenator; LVAD: left ventricular assist device; RVAD: right ventricular assist device.

**Table 4:** 90-day mortality risk score. (**A**) Risk Score; (**B**) RISK PERCENTAGES for EACH SCORE.

(**A**)				
Risk Score				
1	AGE ≥ 67	3	point(s)	
2	55 ≤ AGE < 67	2	point(s)	
3	50 ≤ AGE < 55	1	point(s)	
4	Prior Cardiac Surgery	1	point(s)	
5	Concomitant RV support	5	point(s)	
6	Pre ECMO	2	point(s)	
7	Concomitant surgery	1	point(s)	
			SCORE	
(**B**)				
RISK PERCENTAGES for EACH SCORE				
Score	Risk (%)	Score		Risk (%)
0	3.60%	7		32.50%
1	5.10%	8		41.00%
2	7.20%	9		50.00%
3	10.00%	10		59.00%
4	13.80%	11		67.50%
5	18.80%	12		75.00%
6	25.00%			

**Table 5:** (**A**) Kaplan–Meier Univariable factors influencing long-term mortality. (**B**) Multivariable Cox regression variables influencing long-term mortality. (**C**) Variables used for long-term survival prediction. LVAD-Left Ventricular Assist Device; RVAD-Right Ventricular Assist Device; ECMO Extra Corporeal membrane Oxygenation; IABP-Intraoperative Aortic Balloon Pump; GI-Gastro Intestinal; CVA-Cerebro Vascular Accident.

(**A**)				
Variables	Number of patients		% of patients	*p* value
Preoperative variables				
Every year increase in age				<0.001
Previous cardiac surgery	7763		34.6	<0.001
LVAD exchange	622		2.8	<0.001
ECMO support	1009		4.5	<0.001
Intraoperative variables				
Device type				
LVAD only	20,636		91.9	
RVAD only	30		0.1	0.20
LVAD and temporary RVAD	1335		5.9	<0.001
total artificial heart	466		2.1	<0.001
Concomitant cardiac surgery	4868		21.7	0.001
Postoperative variables				
RVAD support	191		0.9	0.001
Reoperation for bleeding > 48 h	842		3.7	<0.001
Reoperation for bleeding < 48 h	888		4.0	<0.001
ECMO support	785		3.5	<0.001
IABP	5376		23.9	<0.001
Prolonged ventilation	1704		7.6	<0.001
GI bleed	4855		21.6	<0.001
Stroke	3450		15.4	<0.001
Long-term Hemodialysis	4189		18.6	<0.001
(**B**)				
Variables	Hazard Ratio (HR)	Lower bound (95% CI)	Upper bound (95% CI)	*p*-value
Preoperative variables				
Every year increase in age	1.019	1.017	1.021	<0.001
LVAD exchange	1.986	1.231	3.203	0.005
ECMO support	1.400	1.254	1.563	<0.001
Previous cardiac surgery	1.246	1.187	1.308	<0.001
Intraoperative variables				
Concomitant cardiac surgery	1.082	1.025	1.142	0.004
Postoperative variables				
Stroke (CVA)	1.876	1.782	1.974	<0.001
RVAD support	1.613	1.334	1.951	<0.001
Long-term Hemodialysis	2.676	2.548	2.810	<0.001
(**C**)				
Variable	Hazard Ratio (HR)	HR 95% Lower Bound	HR 95% Upper Bound	*p* value
Age	1.022	1.020	1.025	<0.001
Previous Cardiac Surgery	1.25	1.18	1.32	<0.001
Sex (Male = 0, Female = 1)	1.09	1.02	1.16	0.01
Planned Concomitant RVAD	2.25	2.07	2.45	<0.001
Pre ECMO	1.35	1.20	1.52	<0.001
Concomitant Cardiac Surgery	1.06	1.00	1.13	0.04
IABP	1.11	1.04	1.17	<0.001
Stroke (CVA)	1.81	1.71	1.92	<0.001
Post Dial	2.64	2.50	2.78	<0.001

Concordance index: 0.72 on training set, 0.70 on test set.

## Data Availability

Data used to generate this paper will be available on request.

## Abbreviations

UNOS	United Network for Organ Sharing
LVAD	Left ventricular assist Device
INTERMACS	Interagency Registry for Mechanically Assisted Circulatory Support
ECMO	Extracorporeal Membrane Oxygenation
IABP	Intra Aortic Balloon Pump
RVAD	Right Ventricular Assist Device
EUROMACS	European Registry for Patients with Mechanical Circulatory Support
HMRS	Heart Mate Risk Score
HM3RS	HeartMate 3 Risk Score
MOMENTUM 3	Multicenter Study of MagLev Technology in Patients Undergoing Mechanical Circulatory Support Therapy With HeartMate 3
